# Dopamine, Serotonin, and Structure/Function Brain Defects as Biological Bases for Treatment Response in Delusional Disorder: A Systematic Review of Cases and Cohort Studies

**DOI:** 10.3390/bs11100141

**Published:** 2021-10-19

**Authors:** Armand Guàrdia, Alexandre González-Rodríguez, Mary V. Seeman, Aida Álvarez, Francesc Estrada, Sidharta Acebillo, Javier Labad, José A. Monreal

**Affiliations:** 1 Department of Mental Health, Mutua Terrassa University Hospital, University of Barcelona, 08221 Terrassa, Spain; aguardia@mutuaterrassa.cat (A.G.); jamonreal@mutuaterrassa.cat (J.A.M.); 2 Department of Psychiatry, University of Toronto, Toronto, ON M5S 1A1, Canada; mary.seeman@utoronto.ca; 3 Department of Mental Health, Parc Tauli University Hospital, 08280 Sabadell, Spain; aalvarez@tauli.cat (A.Á.); FEstrada@tauli.cat (F.E.); SAcebillo@tauli.cat (S.A.); 4 Department of Mental Health, Consorci Sanitari del Maresme, Fundació Parc Taulí, CIBERSAM, 08340 Mataró, Spain; jlabad@csdm.cat; 5 Neurosciences Institute, Universitat Autònoma de Barcelona, CIBERSAM, 08221 Terrassa, Spain

**Keywords:** delusional disorder, dopamine, neurobiology, psychosis, serotonin

## Abstract

Although blockade of dopamine receptors D2 and D3 appears to be the main mechanism of antipsychotic action, treatment response variability calls for an examination of other biological systems. Our aim is to systematically review reports of treatment response in delusional disorder (DD) in order to help determine its biological bases. Computerized searches of ClinicalTrials.gov, PubMed, and Scopus databases (from 1999 to September 2021) were systematically reviewed, in keeping with PRISMA directives. We used the search terms: (treat * OR therap * AND (delusional disorder)). We included all studies that explored the biological mechanisms of treatment response in DD, as diagnosed by ICD or DSM criteria. A total of 4344 records were initially retrieved, from which 14 papers were included: case reports, case series, and cohort studies. Findings point to (1) dopaminergic dysfunction (based on biochemical and genetic studies), (2) serotonergic dysfunction (based on partial agonism/antagonism of drugs), and (3) brain structure/function impairment, especially in the temporal and parietal lobes, as crucial factors in treatment response. Further studies with higher levels of evidence are needed to help clinicians determine treatment.

## 1. Introduction

Delusional disorder (DD) is classified as a psychosis, a psychiatric condition characterized by reality being experienced in an aberrant way. DD is diagnosed when persistent delusional beliefs last for at least one month. These beliefs are usually not accompanied by prominent hallucinations, and moderately good functioning is, for the most part, preserved [1,2]. The population prevalence of DD is estimated at approximately 0.2% [2], with an incidence of 0.7 to 3.0 per 100,000 [3,4]. DD begins relatively late in life [5]. DSM-5 [2] reports no major gender differences in the demographics of DD; however, in elderly populations, the condition is seen more often in women than in men [6,7]. The Diagnostic and Statistical Manual of Mental Disorders, Fifth Edition (DSM-5) recognizes seven subtypes, divided according to delusional theme [2]. Several comorbid conditions have been identified, depression being the most common [8,9]. Response to treatment is generally seen as poor [10]. Age at onset of the disorder and treatment delay have been hypothesized to affect outcome [2,11].

In contrast, a related condition, schizophrenia, is characterized by disturbances not only of thought, but also of perception and behaviour. As well as delusions, this disorder is marked by auditory and other sensory hallucinations, speech and behaviour disturbances, social withdrawal, apathy, aboulia or restricted affect, and significant impairments in social and cognitive function [12]. According to the Diagnostic and Statistical Manual of Mental Disorders, fifth edition (Diagnostic and Statistical Manual of Mental Disorders -5), the lifetime prevalence of schizophrenia is approximately 1% [2]. The onset of symptoms generally takes place during adolescence and early adulthood. The course of illness tends to alternate between symptom remission and symptom relapse, eventually, in many cases, leading to a significant deterioration of overall function.

Patients with schizophrenia exhibit marked variations in symptoms, symptom domains have no clear biological signature, and responses to therapeutic interventions profoundly vary [13]. Clinical experience indicates that approximately one-third of patients fail to respond to standard antipsychotic treatment. A percentage of such patients do respond to a particular antipsychotic medication, namely clozapine [14,15,16].

Attempts have been made to understand all psychosis through the lens of the dopamine hypothesis, which posits an excess of dopamine and/or an unusually high sensitivity to dopamine as symptom triggers [17]. This hypothesis was first formulated by a Dutch pharmacologist Van Rossum in 1966 [18], after Carlsson and Lindqvist in 1963 implicated monoamine receptors in the mechanism of antipsychotic action of both chlorpromazine (a phenothiazine) and haloperidol (a butyrophenone) [19,20].

Antipsychotic drugs are currently considered the gold standard in the treatment of psychosis, the specific mechanisms of action of these drugs initially thought to derive from dopamine D2/D3 receptor blockade [21,22,23] as well as well as an effect on dopamine synthesis, release capacity, and reuptake in striatal dopamine neurons [24]. Dopamine reuptake depends on the dopamine transporter (DAT).

Increasingly, glutamatergic, serotonergic, and gamma-aminobutyric acid (GABA) -ergic neurotransmitters are being implicated in psychosis. Hypofunction of the glutamate or N-Methyl-D-aspartate (NMDA) receptor in the prefrontal cortex is considered a putative cause of psychosis, as is hyperfunction of the cortical serotonin/5-hydroxytryptamine (5-HT2A) receptor. Gamma-aminobutyric acid (GABA) signalling may play a role since it can lead to aberrant functioning of interneurons, with subsequent dysfunction of cognition and behaviour [13,25]. In schizophrenia, dopamine, glutamate, serotonin, and GABA are all suspected of playing their part in drug response.

With respect to DD, however, there is a paucity of scientific data on treatment response and its causes. Treatment decisions have been made on the basis of results of case series or of observational studies or by making analogies to schizophrenia [26]. Dopamine and serotonin pathways have been held responsible for antipsychotic response [27]. DAT dysfunction has been implicated because DAT inhibitors have been shown to induce psychotic delusions [28,29]. Serotonin or 5-hydroxytryptamine (5-HT2) was implicated in 1990 [30] in monosymptomatic hypochondriasis. The two hypotheses, serotonin and dopamine, were linked because 5-HT1A and 5-HT2A-receptor agonists increased striatal dopamine release [30].

With respect to treatment, starting in the late 1980s, the antipsychotic drug, pimozide, was considered to be the gold standard medication for treatment of the somatic subtype of DD (DDST) [31]. This arose from reports of successful outcomes in a subgroup of DDST, delusional parasitosis (or delusional infestation) [31], even though antidepressants also showed efficacy in this DD subtype [32]. In particular, the antidepressant, clomipramine, was effectively used in patients who failed to respond to pimozide. As a result of the success of clomipramine, which is both a tricyclic antidepressant and a serotonin–norepinephrine reuptake inhibitor, serotonergic dysfunction is now considered a potential risk factor for DDST [30,33].

Neuroimaging studies that visualize brain structure and function under test conditions may be of help in elucidating the underlying neurobiological reasons why some patients show inadequate treatment response, and may be able to identify such patients early. Several brain morphological changes have been associated with positive and negative response to antipsychotic medications in schizophrenia [34,35].

While research into treatment response in schizophrenia has proceeded relatively quickly, it is only just beginning in delusional disorder (DD).

To date, there has been no conclusive evidence supporting a specific biological basis for treatment response in DD. Nevertheless, we hypothesized that a dopaminergic and serotonergic dysfunction, in association with structural and/or functional brain impairment discernible on imaging studies may be responsible for treatment response in DD. Thus, the goal of this review is to examine the literature, looking to strengthen or disprove this hypothesis.

## 2. Materials and Methods

### 2.1. Search Strategy

A.G. and A.G.-R. independently conducted a systematic computerized search on ClinicalTrials.gov, PubMed and Scopus electronic databases from 1999 to September 2021, in keeping with the Preferred Reporting Items for Systematic Reviews and Meta-Analyses (PRISMA) directives [36]. The following search terms were used: ((treat * OR therap *) AND delusional disorder). Additionally, reference lists and book chapters from the studies initially included were hand checked by A.G.R. to identify additional relevant studies. We attempted, with variable success, to contact study authors to obtain further information whenever reported data were incomplete.

### 2.2. Inclusion Criteria

Publications were only included if they met our eligibility criteria: (1) all described cases were adult patients who fulfilled ICD-10 or DSM-5 diagnostic criteria for DD and were receiving psychotropic drugs, (2) publication was in a peer-reviewed journal, (3) the language of the report was English, German, or Spanish, and (4) the case or study tested a biological hypothesis underlying treatment response in DD. The following reports were excluded: (a) those whose participants had clearly developed DD as a result of an organic disease or an injury and (b) those where delusions expressed by patients were clearly secondary to psychiatric diagnoses other than DD. Response to treatment was defined as psychotic symptom remission or marked improvement as measured by appropriate scales, or as derived from physician assessment in clinical records. Most reports found were of single or serial cases where response was determined clinically.

### 2.3. Data Collection and Extraction

**Figure 1:**
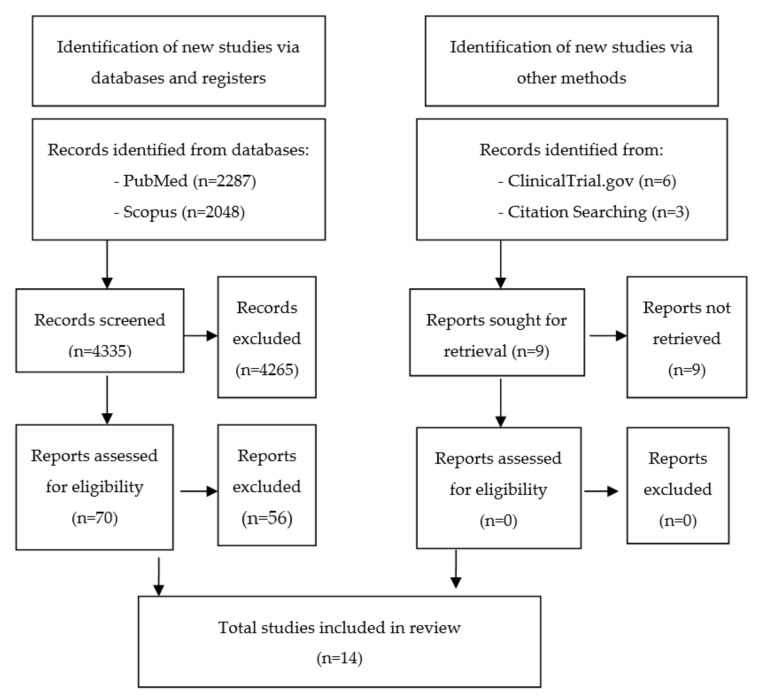
Flow diagram of included reports.

The literature search strategy, data collection, and extraction were conducted independently by A.G.D. and A.G.R. All abstracts and titles were scanned by both authors and manual searches were conducted in references lists of included articles, in order to identify further relevant publications. Disagreement between researchers was resolved by discussion (Figure 1). Information from the selected studies was extracted and is presented in tables (Table 1 and Table 2), text, and Figure 1. The population, intervention, comparator, and outcome (PICO) framework used for this systematic review is detailed in Table A1.

### 2.4. Assessment of Risk of Bias in Included Reports and Quality of Body of Evidence

Risk of bias was evaluated using Case Report Guidelines (CARE) [37] and Tool of Risk of Bias in Case Control Studies devised by the CLARITY Group at McMaster University [38]. Both instruments were used to assess the certainty of the evidence and the strength of the recommendations. Our assessment of the risk of bias is detailed in Table 2, Table 3 and Table A2.

### 2.5. Data Synthesis

Meta-analysis could not be performed due to the heterogeneity of methods used to evaluate treatment outcome. Data were grouped according to initial hypothesis and biological explanation of treatment response.

## 3. Results

A total of 4344 publications were identified: 6 in ClinicalTrials.gov, 2287 in PubMed, 2048 in Scopus, and 3 through supplementary sources. After the screening and selection process, a total of 14 studies that met our criteria were included. Three explanations for the biological underpinnings of treatment response in DD emerged from these reports: (1) dopaminergic dysfunction (n = 5), (2) serotonergic dysfunction (n = 11), and (3) irregular brain structure/function as visualized on brain scan (n = 8).

Individual study design, methods, socio-demographic data of participants, clinical features, DD subtype, and treatment are presented in Table 1 and Table 2.

### 3.1. Reports

#### 3.1.1. Case Reports

Wada et al. [39] reported four cases of patients with DD, somatic type (DDST), who received treatment with clomipramine 60–120 mg/day. Clomipramine was effective for some of the patients with DDST, even for those resistant to pimozide and this was attributed to its serotonergic action. Wada et al. [40] also published another case report of a 78-year-old woman with DDST successfully treated with clomipramine 100 mg/day. The single photon emission computed tomography (SPECT), done when the patient still suffered from profuse hypochondriacal delusion, showed markedly reduced rCBF in the temporal and parietal lobes, most prominent in the left hemisphere. After treatment, rCBF reduction was lessened in the left temporal and parietal lobes. This suggests serotonergic involvement.

Ota et al. [41] described a 79-year-old man with DDST who showed remarkable improvement of clinical symptoms after receiving treatment with modified electroconvulsive therapy (mECT). The Beck Depression Index and Hamilton Depression Rating Scale was used to assess concomitant depressive symptoms. Single photon emission computed tomography (SPECT-Tc-99m) showed a dysfunction of left temporal and parietal lobes, mainly on the left side. In parallel with the improvement of the patient’s clinical symptoms, we observed an improvement of regional cerebral blood flow (rCBF) in the left temporal and parietal lobes on SPECT. This study appears to support the utility of mECT for somatic delusions in elderly patients and suggests a possible association of the dysfunction in the left temporal and parietal lobes with the manifestation of somatic delusions.

A case reported by Hayashi et al. [42] described a 77-year-old woman with somatic delirium who initially showed hypoperfusion in the left temporal and parietal lobes on SPECT imaging, which normalized after remission of psychotic symptoms with paroxetine treatment. Additionally, magnetic resonance imaging (MRI) revealed multiple small infarcts in bilateral deep white matter. This report suggests that paroxetine can be effective for DDST. It also supports previous views that this disorder is associated with serotonergic dysfunction and hypoperfusion in the temporal and parietal lobes.

Dimopoulos et al. [43] published a case report of a 51-year-old woman who suffered DDST and was treated with aripiprazole 15 mg/day combined with mirtazapine 90 mg/day. The response to treatment again suggested that serotonin dysfunction was involved in DDST. Efficacy of drug occupancy of 5-hydroxytryptamine or serotonin (5-HT) receptors in DDST is suggested, although aripiprazole is also a partial dopamine agonist.

Bosmans and Verbanck [44] reported the case of a 48-year-old man with DDST who had a total remission of symptoms on olanzapine 10 mg/day. The authors suggested that this was due to the serotonergic action of olanzapine, which is a 5-HT2 antagonist, although it also blocks dopamine receptors.

A case report of a 75-year-old man with delusional parasitosis by Huber et al. [45] hypothesized a dysfunction of the dopamine transporter (DAT) in DDST. The striatum was investigated by magnetic resonance imaging (MRI), and showed no basal ganglia or subcortical gray matter lesions. Nonetheless, the positive treatment effects of risperidone 1–3 mg/day supported a dopaminergic dysfunction in DDST.

Akahane et al. [46] presented a case of a 54-year-old man with gross somatic delusions, which responded to treatment with risperidone. Single photon emission computed tomography (SPECT-Xe-133) showed hypoperfusion of the temporal and parietal lobes (mainly on the left), which normalized after successful risperidone treatment.

A successful response of DDST to paroxetine in a 42-year-old woman who was also depressed was reported by Hayashi et al. [47]. They described a first treatment response to olanzapine 10 mg/day, later switched to paroxetine 20 mg/day. Prior to treatment, they observed reduced cerebral blood flow in the left temporal and parietal lobes. After treatment, the hypoperfusion in the temporal and parietal lobes was normalized. The report implies that paroxetine, through its serotonergic action, was effective for DDST.

In a case report of multimodal imaging of a 27-year-old woman with delusional parasitosis, Freudenmann et al. [48] found altered pre- and postsynaptic dopaminergic neurotransmission in the striatum, mainly the left putamen. Additionally, glucose metabolism was left-dominant in the thalamus and putamen. Full remission after treatment with aripiprazole was associated with 63% to 78% striatal D2 receptor occupancy and glucose metabolism changes in both thalami. The conclusion was that partial antagonism of DRD2 was responsible for the improvement. This case suggests that the fronto-striato-thalamo-parietal network, brain regions involved in judgment, sensory gating, and body perception, may be involved in producing the core symptoms of delusional infestation (DI).

Rajkumar et al. [49] reported on a 44-year-old woman with DD who presented with supersensitivity psychosis due to ziprasidone 100 mg/day. The patient improved after treatment was switched to asenapine 20 mg/day. The conclusion was that ziprasidone-induced worsening of psychotic symptoms and perioral and lingual dyskinetic movements may be explained by chronic blockade of dopamine receptor 2 (DRD2) and that this, therefore, was the likely mechanism responsible for psychotic symptoms.

Davis and Agarwal [50] published the first case report of a 41-year-old woman with DD with persecutory delusions who was successfully treated with lurasidone 120 mg daily. Lurasidone acts through dopamine D2 and serotonin 5-HT2A receptor antagonism. It also shows some partial agonist action at 5-HT1A receptors and antagonism at 5-HT7 receptors, which may be beneficial for mood, anxiety, and cognition in a number of disorders, including delusional disorder.

Two cases of oral somatic delusions (a 73-year-old man and a 72-year-old woman) that responded well to aripiprazole were reported by Umezaki and colleagues [51]. The Oral Dysesthesia Rating Scale (Oral DRS), the Symptom Severity Scale (SSS), and the Functional Impairment Scale (FIS) were used to identify psychopathological symptoms. The first case improved with aripiprazole 1.5 mg/day and the second improved after receiving a combination of aripiprazole 1.5 mg/day and mirtazapine 45 mg/day. The authors concluded that low-dose aripiprazole improved the symptoms, suggesting that both dopaminergic (through DRD2 partial agonism) and serotonergic systems may be involved in the pathology of oral cenesthopathy or oral dysesthesia, a form of somatic DD. These two patients also had SPECT images done, which suggested to the authors that right > left rCBF asymmetry in the frontal and temporal lobes and thalamus as well as dopaminergic and serotonergic dysfunctions are involved in the pathology of oral cenesthopathy. In both cases, the asymmetric rCBF patterns were attenuated after successful treatment.

Table 1 summarizes all studies classified according to the hypotheses they address, Table 2 reports main characteristics of the included studies, and Table 3 presents risk of bias of included reports.

**Table 1:** Hypotheses addressing the biological basis of antipsychotic response in delusional disorder.

(1) Dopaminergic dysfunction (‘dopamine psychosis’) (n = 5)	
	1- Ziprasidone induced-supersensitivity psychosis by chronic blockade of DRD2 in mesolimbic brain [49].
	2- Pretreatment levels of pHVA and implication of DRD2 Ser311Cys, DRD3 Ser9Gly and TH VNTR in DD [52]
	3- DAT dysfunction in DDST [45]
	4- Effectiveness of DRD2 partial agonists (aripiprazole) [48,51].
**(2) Serotonergic dysfunction (n = 11)**	
	1- Efficacy of partial agonism 5-HT1A and antagonism 5-HT2A in DDST [43,48,50,51].
	2- Efficacy of 5-HT2 antagonists in DDST [39,40,42,43,44] and DD [49].
	3- Efficacy of partial agonist at 5-HT_1A_ and 5-HT7 antagonists in DD [50].
**(3) Brain dysfunction (n = 8)**	
	1- Serotonergic and dopaminergic reversal of reduced rCBF in left temporal and parietal lobes in DD [40,41,42,46,47].
	2- Serotoninergic and DRD2 partial agonistic reversal of reduced rCBF in right temporal and parietal lobes in DD [51].
	3- DRD2 partial antagonistic reversal of dysfunctional fronto-striato-thalamo-parietal network [41].
	4- Correction of basal ganglia and subcortical grey matter lesions correlates with good response in DD [48].

**Table 2:** Main characteristics of reports addressing the biological basis for treatment response in delusional disorder (n = 14).

Authors and Year of Publication	Study Design	Method	Checklist CARE Guidelines	
			Completed Items	Missing Subitems *,^#^
Umezaki et al., 2017 [51]	Case reports	Neuroimaging: 99m Tc ECD SPECT	7/13	2, 5c, 8a, 8d, 10c, 10d, 12, 13
Davis and Agarwal 2015 [50]	Case report	Clinical observation	6/13	2, 3b, 3c, 6, 8a, 8b, 8d, 10a, 10b, 10d, 12, 13
Rajkumar et al., 2014 [49]	Case report	Clinical observation	6/13	2, 3d, 6, 8a, 8b, 8d, 10a, 10c, 12, 13
Freudenmann et al., 2010 [48]	Case report	Neuroimaging: (a) Untreated state: FDOPA-PET, 123I-FP-CIT-SPECT, IBZM-SPECT and FDG-PET (b) After AP antipsychotic treatment: IBZM-SPECT, FDG-PET	6/13	1, 2, 5a, 5c, 8b, 8d, 10a, 10c, 10d, 12, 13
Hayashi et al., 2010 [47]	Case report	Neuroimaging: 99m- Tc ECD SPECT	5/13	1, 2, 5a, 5c, 8b, 8d, 9b, 9c, 10a, 10c, 10d, 12, 13
Akahane et al., 2009 [46]	Case report	Neuroimaging: SPECT-Xe-133	5/13	2, 5a, 5c, 6, 8b, 8d, 9c, 10a, 12, 13
Bosmans and Verbanck, 2008 [44]	Case report	Clinical observation	4/13	1, 2, 3a, 3b, 3c, 3d, 5a, 5c, 8b, 8d, 9b, 9c, 10a, 10c, 12, 13
Huber et al., 2008 [45]	Case series (consecutive sampling)	Neuroimaging: MRI (T1, T2, FLAIR)	6/13	1, 2, 5c, 8b, 10a, 12, 13
Dimopoulos et al., 2008 [43]	Case report	Clinical observation	4/13	1, 2, 3b, 5c, 6, 8b, 10a, 10c, 10d, 12, 13
Hayashi et al., 2004 [42]	Case report	Neuroimaging: MRI; Xe-133 SPECT	5/13	1, 2, 5a, 5c, 8b, 8d, 9c, 10a, 10c, 10d, 12, 13
Ota et al., 2003 [41]	Case report	Neuroimaging: MRI, MRA, 99m- Tc ECD SPECT	6/13	2, 5c, 5d, 8b, 8d, 9b, 9d, 10a, 10c, 10d, 12, 13
Morimoto et al., 2002 [52]	Prospective observational cohort study	(a) Biochemistry: p HVA (HPLC) (b) Genetics: polymorphisms of DRD2 Ser311Cys, DRD3 Ser9Gly, TH VNTR (c) Clinical: AP response	-	-
Wada et al., 1999 (b) [40]	Case report	Neuroimaging: 133-Xe SPECT + MRI	4/13	1, 2, 5a, 5c, 6, 8b, 8d, 9c, 10a, 10c, 10d, 12, 13
Wada et al., 1999 (a) [39]	Case report	Clinical observation	0/13	1, 2, 3a, 3b, 3c, 3d, 4, 5a, 5c, 5d, 6, 7, 8a, 8b, 8d, 9b, 9c, 9d, 10a, 10b, 10c, 10d, 11a, 11b, 11d, 12, 13

* Checklist items from CARE guidelines include: 1, 2, 3a, 3b, 3c, 3d, 4, 5a, 5b, 5c, 5d, 6, 7, 8a, 8b, 8c, 8d, 9a, 9b, 9c, 10a, 10b, 10c, 10d, 11a, 11b, 11c, 11d, 12, 13. ^#^ Items that are not applicable for the case report are not included in this section.

**Table 3:** Risk of bias assessment for studies included in the Systematic Review (n = 13) [37].

Tools for Evaluating Methodological Quality of Case Reports														
Domains	Leading explanatory questions	[39]	[40]	[41]	[42]	[43]	[44]	[45]	[46]	[47]	[48]	[49]	[50]	[51]
Selection	1. Do the patient(s) represent the whole experience of the investigator or is the selection method unclear to the extent that other patients (…)?	No	No	Yes	Yes	Yes	No	Yes	No	Yes	Yes	No	Yes	Yes
Ascertain-ment	2. Was the exposure adequately ascertained?	Yes	Yes	Yes	Yes	Yes	Yes	Yes	Yes	Yes	Yes	Yes	Yes	Yes
	3. Was the outcome adequately ascertained?	No	Yes	Yes	Yes	Yes	Yes	Yes	Yes	Yes	Yes	Yes	Yes	Yes
Causality	4 *. Were other alternative causes that may explain the observation ruled out?	No	No	No	No	No	No	No	No	No	No	Yes	No	No
	5 *. Was there a challenge/rechallenge phenomenon	No	No	No	No	Yes	No	No	No	Yes	No	Yes	No	Yes
	6 *. Was there a dose-response effect?	Yes	Yes	No	Yes	Yes	Yes	Yes	Yes	Yes	Yes	No	Yes	Yes
	7. Was follow-up long enough for outcomes to occur?	Yes	Yes	Yes	Yes	Yes	Yes	Yes	Yes	Yes	Yes	Yes	Yes	Yes
Reporting	8. Is the case(s) described with sufficient details to allow other investigators to replicate the research or to allow practitioners make inferences related to their own practice?	No	Yes	Yes	Yes	Yes	Yes	Yes	Yes	Yes	Yes	No	Yes	Yes
Total scores	Max. 8	5	6	6	7	7	6	7	6	7	7	6	7	7

* Questions 4–6 are particularly relevant for cases reporting adverse drug events. Total scores are an overall judgement about methodological quality and not the total sum of the 8 items.

#### 3.1.2. Trial Addressing the Dopamine Hypothesis of Drug Response in DD

A report by Morimoto et al. [52] consists of three separate studies, two of which are pertinent to this review. In this prospective observational cohort study, the Brief Psychiatric Rating Scale (BPRS) was used to evaluate response to treatment. The first study set out to assess response to antipsychotic medication in DD patients versus schizophrenia patients diagnosed using ICD10 criteria. Both groups were first episode patients and were drug naïve. The mean age of the 11 DD patients was 57.5; only one of the 11 was a male. The mean age of the 15 schizophrenia patients was 25.1, 7 males and 8 females. Outcome measures of response were mean effective haloperidol dose, duration of admission, and Global Assessment of Functioning (GAF) score at discharge. The results showed that DD patients achieved remission of symptoms, on average, within 65 days on 4.7 mg/d of haloperidol while patients with schizophrenia required 12.7 mg/d for 104 days. GAF score at discharge were 10 points higher in DD than in schizophrenia patients. In summary, the DD first episode group, mostly female and over 50 years of age, appeared to have a significantly more robust response to haloperidol than young adults of both sexes diagnosed for the first time with schizophrenia. The second relevant Morimoto et al. [46] study examined the initial levels of plasma homovanillic acid (pHVA), a dopamine metabolite and clinical marker of dopamine metabolism, in 13 drug naïve in and out patients with DD, whose mean age was 48. Four were men and 9 were women; 9 suffered from persecutory delusions and 4 from delusional jealousy. The BPRS was used to assess symptoms. Levels of pHVA were compared with those of healthy controls matched for age and sex. In 8 of the DD patients, pHVA levels were also obtained after 8 weeks of treatment with haloperidol. The results of the pre-treatment pHVA levels were that DD patients had levels that were almost twice those of controls. Furthermore, the levels correlated positively with BPRS scores on psychotic symptoms. Looking further into the results, the investigators noticed that DD patients with persecutory delusions were wholly responsible for the results. The pHVA levels of those with delusional jealousy were equivalent to those of controls. After treatment (average haloperidol dose 2.7 mg/d), 5 of the 8 patients with persecutory delusions had achieved complete remission and there was a significant group decrease in pHVA compared to pretreatment levels. The authors concluded that DD, especially the persecutory subtype, was a “dopamine psychosis” but that further research was necessary before this could be stated with any certainty.

## 4. Discussion

Our review shows that very few studies have investigated the mechanism of drug response in DD. The majority of our citations are case reports, which help to pose questions and develop hypotheses, but are not in themselves evidence and do not allow population-based inferences. Interestingly, no case studies have suggested biological hypotheses other than the ones we initially proposed.

With respect to the dopamine hypothesis, the implication of dopamine transporter (DAT) dysfunction in DD is supported by some case reports [45,53] showing that DAT-inhibitors, (cocaine, pemoline, methylphenidate, and other amphetamine-derivatives) can induce delusional symptoms [28,29]. This is consistent with the theory that increased levels of extracellular dopamine in the striatum of DD patients (at least in those with the somatic subtype) may result from decreased DAT-functioning rather than increased rates of dopamine release [53].

The blockade of D2 and D3 receptors [54,55,56] is also potentially supported by these literature reports, consistent with dopamine antagonists being the treatment of choice for DD [57].

In line with reports of occupancy of 5-HT1A and 5-HT2A receptors correlating with treatment response in DD [49,50], King and colleagues in 1990 [30], taking response to lysergic acid diethylamide (LSD) as a model, had previously discussed a serotonergic hypothesis for the clinical expression of monosymptomatic hypochondriasis (including delusional parasitosis). They theorized that 5-HT1A and 5-HT2A-receptor agonists (such as psilocybin for instance) increased striatal dopamine release. The results of our literature search indirectly support the hypothesis of a serotonin-dopamine dysregulation in DD and help to explain the fact that antidepressants as well as antipsychotics show efficacy in this disorder [25,58].

Co-morbid mood disorder is estimated to occur in 32 to 53 percent of all delusional disorder patients [59]. Since serotonin 5-HT2A and 5-HT7 receptor antagonism would be expected to lift comorbid depression [9] and, thus, improve the patient’s overall state, it therefore cannot be concluded that DD without depression would also respond to this mechanism of drug action. The available literature does not shed light on this question. While high rates of comorbid depression have been classically described in patients with DD, the issue of whether depressive symptoms are comorbid or part of the psychopathological construct of DD remains controversial. In contrast with studies reporting comorbid depression rates in DD, Serretti and collaborators [60] consider depressive symptoms to be a core part of the structure of DD. These investigators analysed the symptomatic domains of DD using the Operational Criteria (OPCRIT) checklist for psychotic illness. They identified four main factors: (1) delusions, (2) irritability symptoms, (3) hallucinations, and (4) core depressive symptoms. Moreover, de Portugal et al. [61] carried out a cross-sectional study in 86 outpatients with DD and conducted a factor analysis using scores on the Positive and Negative Syndrome Scale (PANSS). They identified four independent psychopathological dimensions in DD: (1) paranoid, (2) cognitive, (3) schizoid, and (4) affective. In their report, the affective dimension was associated with a family history of depression, a risk for suicide, and relatively high perceived stress. The issue of the role of depression in DD requires further investigation.

Our review found 8 reports of structure/function defects on brain imaging that appeared to predict treatment response [40,41,42,45,46,47,48,51]. Several brain morphological changes have, in the past, been associated with positive and negative response to antipsychotic medications [34,35].

These studies demonstrate that brain imaging irregularities recede or disappear in those who respond to treatment and remain in those who do not. Following this line of reasoning, structural and functional magnetic resonance imaging could provide information on potential neural substrates of the disease.

Because of the relatively late age of the DD population, future studies need to control for age and disease duration. The vast majority of reports do not provide these data. The main neuroimaging findings associated with response appear, from our search, were hypoperfusion in the temporal and parietal lobes, mainly on the left, because these normalize after successful treatment.

Lesions of the basal ganglia and subcortical gray matter also resolve with a good response. Furthermore, dysfunction in the fronto-striatal-thalamus-parietal network (brain regions involved in judgement (frontal cortex), body perception (dorsal “loop” and parietal “somatic” cortices), and sensory gating (thalamus) and percentage occupancy of D2 striatal receptors also reflect clinical response to antipsychotics in DD.

Reports of brain irregularities in psychiatric conditions are difficult to interpret. They are informative if they disappear with symptom remission [62]. When treatment is unsuccessful and brain impairment persists, it is impossible to know whether the fault lies in the treatment or whether the presence of the impairment preceded the disorder. There is insufficient information in the scientific literature to answer these questions.

Important to the discussion of our literature findings is the relative lack of consensus about the operational definition of response to treatment in DD. An agreed upon definition would need to be determined before investigations of biological mechanisms of treatment response could be accurately interpreted. Additionally, it is important to understand that, despite the many similarities of DD with schizophrenia, several factors suggest that delusional disorder is an independent disorder—it starts later in life than schizophrenia, negative and cognitive symptoms are rarely present, auditory hallucinations are rare, and, for the most part, adequate functioning is maintained [63,64,65,66]. Because of its late age onset, there is a potential overlap in DD with neurologic disorders of older age, a significant issue with respect to treatment response. Organic delusional disorders have been observed in a wide variety of neurological disorders [67]. Delusion is associated with Parkinson’s disease, stroke, and right hemisphere lesion in the temporo-parietal zones or subcortical areas [68,69]. Very late first-contact delusional disorder is known to increase by 5–8 times the risk of subsequent dementia compared with the general population [70].

Treatment resistance in DD has not been studied in the way that it has in schizophrenia. For instance, the chief drug for treatment resistance in schizophrenia, clozapine, is rarely used in DD [71]. Because of the significant presence of brain defects and neurological problems in DD, hypotheses of response mechanisms borrowed from schizophrenia studies may not apply.

### Limitations and Strengths

Our conclusions are limited by the fact that very few reports exist that specifically address a possible mechanism for therapeutic response in DD. Even among the few we found, the conclusions did not necessarily agree with each other. The number of subjects reported on is very limited and case reports can only be suggestive. The possibility of publication bias also exists, clinicians making the assumption, perhaps, that what applies to schizophrenia must also apply to DD and reporting only positive results. The cases we cite mainly refer to chronic patients with varying duration of untreated psychosis and heterogeneous medication histories. Psychometric assessment tools also differ, although all the scales currently used are considered comparable.

While a variety of neurotransmitters are now implicated in drug response in schizophrenia [72,73], in DD, there are no reports on drug response involving glutamatergic or GABAergic neurotransmitter pathways. More research is needed on these pathways in DD.

Despite the lack of large randomized double blind trials, the reports we cite show a low risk of bias and the cases cited contain a great deal of information. They generate hypotheses that can be tested in future studies. This is a strength of our systematic review, the first to summarize clinical experience that sheds light on the biological underpinnings of treatment response in DD. Our hope is that it will help clinicians determine optimal treatment for this population.

## 5. Conclusions

Non-response to antipsychotic drugs remains a significant clinical problem in the treatment of patients with DD. This condition has been understudied; its treatment relies heavily on case reports and case series because scientific data and clinical guidelines are not yet available. Antipsychotics are generally favored as the most effective method of treatment of DD, but antidepressants have also proven useful. It may be that some delusions are secondary to depression and that treating depression indirectly reduces the need for elaborating a delusion. There is a psychological component to delusional disorder that we have not addressed in this review.

Our literature search yielded mainly case reports; nevertheless, findings suggest that dopaminergic and serotonergic neurotransmission is crucial for drug treatment response in DD and that brain imaging irregularities disappear in patients who respond to treatment while they remain unchanged in those who do not.

To sum up, dopamine, serotonin, and brain irregularities are probably associated with treatment response in delusional disorders. This supports the use of antipsychotics and antidepressants to treat patients with DD but, to improve response, randomized treatment trials of a variety of treatment interventions are indicated. It may be that drugs acting through novel neurotransmitter systems will enhance treatment outcomes.

## Data Availability

The data presented in this review are available on request from the corresponding author.

## Abbreviations

Cys	Cysteine
DAT	Dopamine Transporter
DD	Delusional Disorder
DDST	Delusional Disorder Somatic Type
DRD2	Dopamine Receptor D2
DRD3	Dopamine Receptor D3
Gly	Glycine
pHVA	plasma homovallinic acid
rCBF	regional Cerebral Blood Flow
Ser	Serine
TH	Tyrosine Hydroxylase
VNTR	Variable Number of Tandem Repeat
5-HT	5-hydroxytryptamine

## Appendix A

**Table A1:** PICO scheme of the systematic review.

Patient, Population or Problem	Intervention or Exposure	Comparison	Outcome
What are the characteristics of the patients or population? What is the problem, condition or disease you are interested in?	What interventions are we considering?	What is the comparison or alternative to the intervention?	What are the possible or relevant outcomes?
Patients with delusional disorder (DD). Review studies reporting an hypothesis for the explanation of the biological underpinnings of treatment response in DD or reporting structural or functional neuroimaging findings.	Pharmacological treatment for DD.	Any comparator (placebo, different drugs, any other therapy)	Complete remission or marked improvement of psychotic symptoms in DD. New knowledge about the biology of treatment response in DD.

**Table A2:** Risk of bias assessment for reports included in the Systematic Review (n = 1).

Tools for Evaluating Methodological Quality of Case Controls	
Leading Explanatory Questions	Morimoto et al., 2002 [52]
1. Can we be confident in the assessment of exposure?	Definitely yes
2. Can we be confident that cases had developed the outcome of interest and controls had not?	Probably yes
3. Were the cases (those who were exposed and developed the outcome of interest) properly selected?	Probably yes
4. Were the controls (those who were exposed and did not develop the outcome of interest) properly selected?	Probably yes
5. Were cases and controls matched according to important prognostic variables or was statistical adjustment carried out for those variables?	Probably yes
